# Haptic communication between humans is tuned by the hard or soft mechanics of interaction

**DOI:** 10.1371/journal.pcbi.1005971

**Published:** 2018-03-22

**Authors:** Atsushi Takagi, Francesco Usai, Gowrishankar Ganesh, Vittorio Sanguineti, Etienne Burdet

**Affiliations:** 1 Institute of Innovative Research, Tokyo Institute of Technology, Yokohama, Japan; 2 Department of Bioengineering, Imperial College of Science, Technology and Medicine, South Kensington, London, United Kingdom; 3 Department of Informatics, Bioengineering, Robotics and Systems Engineering, Università degli studi di Genova, Genova, Italy; 4 Department of Psychology and Neuroscience, Dalhousie University, Halifax (NS), Canada; 5 CNRS-AIST JRL (Joint Robotics Laboratory), UMI3218/RL, Umezono, Tsukuba, Ibaraki, Japan; Johns Hopkins University, UNITED STATES

## Abstract

To move a hard table together, humans may coordinate by following the dominant partner’s motion [1–4], but this strategy is unsuitable for a soft mattress where the perceived forces are small. How do partners readily coordinate in such differing interaction dynamics? To address this, we investigated how pairs tracked a target using flexion-extension of their wrists, which were coupled by a hard, medium or soft virtual elastic band. Tracking performance monotonically increased with a stiffer band for the worse partner, who had higher tracking error, at the cost of the skilled partner’s muscular effort. This suggests that the worse partner followed the skilled one’s lead, but simulations show that the results are better explained by a model where partners share movement goals through the forces, whilst the coupling dynamics determine the capacity of communicable information. This model elucidates the versatile mechanism by which humans can coordinate during both hard and soft physical interactions to ensure maximum performance with minimal effort.

## Author summary

Humans are talented at coordinating movements with one another through a multitude of objects such as a hard table or a soft mattress. Depending on the softness of the object, the force we perceive from the partner can be strong enough to sense directional cues, or could be too weak to understand the partner’s movement intention. How do we coordinate physical movements governed by such differing mechanics? Our task is inspired by a pair moving through a dancefloor during Tango dancing; we tested subjects in pairs who jointly chased a moving target with their right hands, which were banded together by either a strong, medium or weak elastic band. By measuring the change in each partner’s performance at the task, and the muscular effort they exerted, we characterized the changes in each partner’s behavior as a function of the strength of the elastic band that coupled them together. By employing a computational simulation of the task, we tested different coordination mechanisms to see what explained the data best. We found that, regardless of the coupling strength, each subject infers the movement intention of their partner, but this process deteriorates with softer coupling.

## Introduction

When moving into a new home, workers help each other to manipulate various objects such as hard tables and soft mattresses. They may verbally agree which room to move towards, but will rely on subtle haptic cues provided by touch and proprioceptive sensing to coordinate their movement through narrow corridors and stairs. Importantly, the mechanics of the object they transport will influence the haptic forces between the partners and may require them to adopt different interaction strategies. In the case of interaction through stiff coupling, previous studies have suggested that one follows the better partner’s lead [1–5]. However, manipulating a soft object like a mattress, which has internal degrees of freedom, may require more complex forms of coordination as one can rely less on the partner to move the object skillfully, and the object’s mechanics influences the perception of the partner’s force [6]. On the other hand, a hard table directly transmits a partner’s mistakes, thereby disturbing one’s motion, while a soft object can be helpful in this situation as the transmitted mistakes are less perturbing. Thus, the interaction strategy is likely to depend on the sensory information available from the interaction forces. Similar situations are encountered during direct physical contact between humans. For example, a therapist moving a patient’s hand would correspond to a *hard interaction*, whilst a parent helping their child to take their first steps is a *soft interaction* as the compliant arms mediate the interaction. To physically interact in such versatile scenarios with different *coupling dynamics*, humans may change their behavior depending on how hard or soft the interaction is, but we do not know whether nor how people do so.

What do we know about such physical interaction between humans? In the past decade, physical coordination has been investigated [2–5,7–13] using a variety of tasks and metrics to investigate the mechanism of physical coordination, but our understanding of the mechanism remains shallow [9,13]. The interactive task can be either discrete [2] or continuous [11], where dyads are coupled directly through stiff mechanics such as a crank [14] or through robotic devices [15]. Previous studies have used several metrics such as the energy exchanged between partners [3], dominance measures [4], distance from a goal [8,9] and magnitude of interaction force [11] to quantitatively analyze physical interaction. For example, a previous study examined stiffly coupled dyads rotating a crank to perform sequential reaching movements [14], and found force patterns of one partner purely accelerating the crank and the other slowing it, which the authors ascribed to the adoption of roles. However, it is not clear if these force patterns were evidence of role emergence or were artefacts of different movement speeds [13,16]. Thus, there is a need to understand how people modify their coordination strategy depending on the coupling strength to the partner.

**Fig 1:**
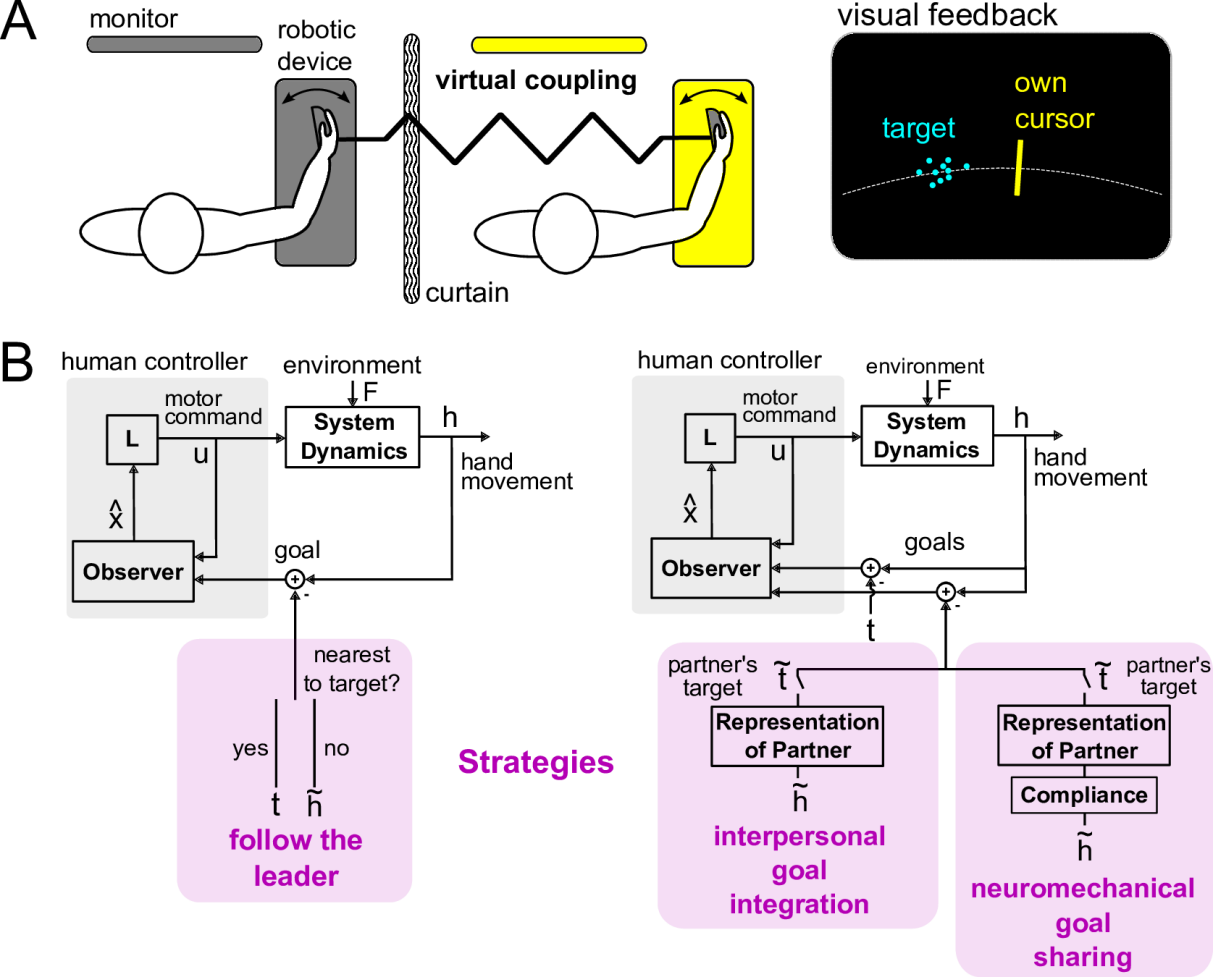
Experimental paradigm and simulation framework to investigate physical interaction between humans mediated by the coupling dynamics. (A) Experimental configuration to test physical interaction in 1 dimension. Subjects, recruited in pairs, were separated by a curtain and followed a common target using their wrist flexion/extension to control a cursor along a 1 dimensional arc. Their wrists were virtually coupled together through the dual robotic interface by a spring with computer-controlled elasticity. Only the target, which was a normally distributed cloud of spots, and one’s own cursor were displayed on each subject’s individual monitor. (B) The computational framework to test different strategies (in purple) that partners could adopt during interaction. In this approach, only the goal or what information is used to track the target is changed. The goal is tracked by a subject who generates a motor command to move their wrist. Two subjects are simulated in parallel, who experience the coupling force from the spring. In *follow the leader*, subjects switch to following the partner nearest to the target. In *interpersonal goal integration*, the partner’s target is inferred through haptics and is integrated with one’s own target estimated from vision. *Neuromechanical goal sharing* is similar to *interpersonal goal integration*, but the weighting between the visual target and the partner’s target is influenced by the coupling dynamics.

To understand how people change their interaction behavior, we developed an experiment that systematically examines how the coupling dynamics affects the performance and the muscular effort of two partners during an interactive task (see Fig 1A). We chose to study physical interaction in a continuous tracking task, where subjects follow a common, randomly moving target using their right hand [17]. Such a continuous task has been shown [18] to cause *coordination* between partners mediated by an exchange of information on the current motion goal through haptics. We developed and tested computational models to explain how the two partners’ performance and effort change with the coupling dynamics (see Fig 1B).

How do partners respond to the different demands of the interaction with differing coupling mechanics? In a previous study, we used a tracking task to show that partners who are compliantly interacting together share their movement goals, which improves both partners’ predictions of the target’s motion, resulting in improved tracking of the target for both partners [18]. This occurs through the optimal sensory integration of information from vision and haptic sensing [19,20], and is also reported to occur between proprioception and force-sensing [6]. Typically, the subject’s uncertainty in each sensory modality is measured. Next, they are given different information for each sensory modality. For example, the experimenter asks subjects to determine the angle of a slanted plane, which can be determined visually or by haptically touching the surface through a robotic interface. The subject is given conflicting visual and haptic information, but their guess is found to be a weighted combination of the two, with the noisier sensory modality weighed less [19]. A similar integration of sensory information was documented in dyads that track a common target whilst compliantly coupled to each other. However, rigidly coupled partners have been suggested to adopt roles [1–4,11] where one partner follows the lead of the more capable partner. In a previous study, we suggested that reaching tasks may be too short for roles to emerge [16], but leadership roles could emerge in a longer interactive tracking task where dyadic coordination was observed [18]. Thus, we hypothesized that hard interactions are governed by a strategy of following the leader or the more capable partner, and compliant interactions can be explained by the sharing of movement goals, as was found in our previous study [18]. We test this hypothesis by examining how a pair or *dyad* tracks a common moving target whilst their hands are coupled by a hard, medium or soft elastic band.

## Results

We recruited 14 pairs of subjects, and asked each dyad to track the same randomly moving multi-sinusoidal target, which was displayed on two individual monitors as 10 circular spots distributed normally around the actual target position (see Fig 1A). Partners controlled their own cursor using their wrist flexion-extension to track this target, and each partner could only see the target and their own cursor. Partners were separated by a curtain throughout the experiment to mitigate biased behaviors due to social factors [21], and were prohibited from verbally interacting. Dyads experienced *solo trials*, where the two subjects tracked the target alone, and *connected trials*, where the dyad were physically coupled by a virtual elastic spring produced by a dual robotic interface [22] during the whole 40 second trial. The solo trials were used as a baseline to assess the effects of physical interaction. Task performance was measured by the *tracking error*, which is defined as the root-mean squared difference between the target and the cursor over the whole trial. Effort in each partner was quantified using surface electromyography of a wrist flexor-extensor muscle pair, with each muscle calibrated with respect to torque (see Experiments for details). The whole experiment consisted of 45 trials that were either solo or connected trials, which was determined by a binary die. The 45 trials were split into three blocks of 15 trials, where in each block the strength of the elastic spring was set such that partners experienced *hard* (*K* = 17.2 Nm/rad), *medium* (*K* = 1.7 Nm/rad) and *soft* coupling dynamics (*K* = 0.3 Nm/rad). All dyads experienced the medium block first, but the order of the soft and hard blocks was randomized. This ordering of the blocks did not influence the results (see Experiments for linear-mixed effects analysis). Furthermore, we observed no learning effect as the solo errors remained constant throughout the experiment (χ^2^(1) = 0.90, *P*>0.34).

**Fig 2:**
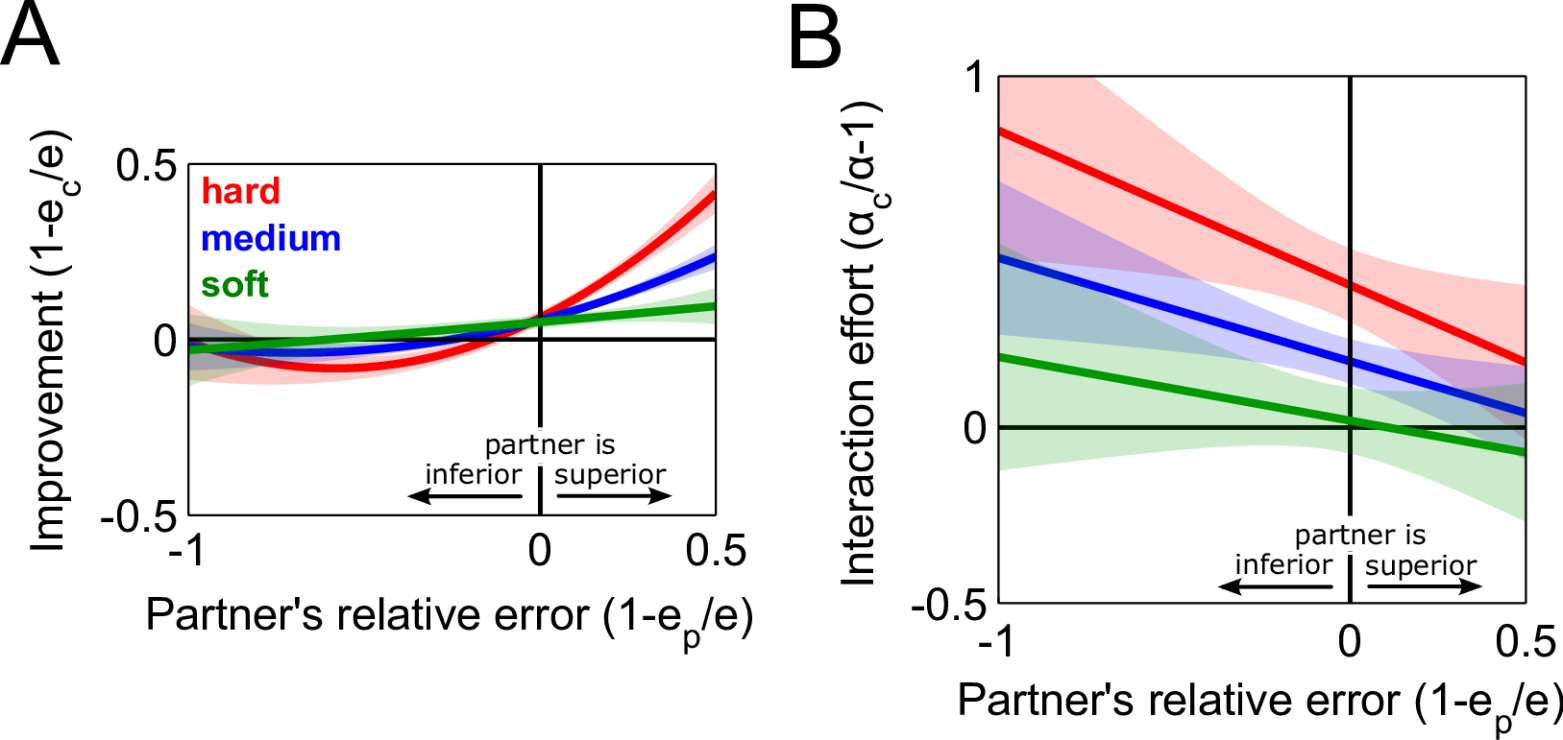
Results of physical interaction between humans through different coupling dynamics. (A) Improvement, defined as the tracking error in a solo trial minus the error in the preceding connected trial, as a function of the relative error, defined as the difference in tracking errors between the partners in the solo trial. The improvement was modulated as a function of the coupling stiffness such that the worse partner (positive in the horizontal axis) improved more with the hard than with the soft interaction. However, the hard interaction did not hinder the better partner’s performance. (B) Interaction effort, defined as the effort expended during a solo trial minus the effort in the preceding connected trial, as a function of the relative error. The effort was estimated as the sum of the mean muscle activations, normalized with respect to torque, from a wrist flexor and extensor pair. As with the improvement, the interaction effort is modulated by the softness of the interaction. Only the better partner in the hard and medium interactions exerted more effort in comparison to solo trials.

First, we examined how the coupling dynamics affected the partners’ tracking errors. We define the *performance improvement* as the change in the tracking error, i.e. the ratio of the difference 1 – *e*_*c*_/*e* between *a subject’s error e* in a solo trial and their error in the previous connected trial *e*_*c*_. Note that this error in the connected trial *e*_*c*_ is similar for both partners during a hard interaction, but can be different for medium and soft interactions. In Fig 2A, the performance improvement is plotted as a function of the relative error 1 – *e*_*p*_/*e*, the difference between a subject’s error and their *partner’s error e*_*p*_ in the same solo trial, enabling us to observe how each subject improved when interacting with a partner who was better or worse at the tracking. The plot of improvement as a function of the relative error is shown for the hard (red trace), medium (blue trace) and soft (green trace) interactions, where the lines come from the data that were fit using a linear mixed-effects model with the improvement as the dependent variable, and with linear and quadratic forms of the relative error and the log of the coupling stiffness as predictors (see Experiments for details). We compared two linear mixed-effects models, one with and the other without the coupling stiffness predictor, and found that the stiffness of the interaction significantly modulates the performance improvement (χ^2^(3) = 67.0, *P*<10^−13^).

To examine how the partners were influenced by the coupling stiffness, we calculated the mean improvement of the better and worse partners, which was determined using their relative error, within each dyad for every level of coupling stiffness. A repeated-measures ANOVA determined that the interaction effect of the better/worse partner by the coupling stiffness significantly changed the mean improvement (*F*(2,44) = 15.5, *P*<0.0012). Post-hoc Tukey-Kramer tests revealed that the better partner’s performance was similar for all coupling strengths, so they were not hindered by the worse partner during the hard interaction. On the other hand, the worse partner’s improvement was greater during hard interaction in comparison to medium (*P*<0.04) and soft interactions (*P*<0.005). Altogether, these results imply that a hard interaction enables a worse partner to achieve the same performance as the better partner, which could be due to the better partner leading the movement to facilitate the task with the worse partner. The worse partner’s improvement came at no cost to the better partner’s performance.

Next, we examined how the partners’ effort was affected by the coupling dynamics. The effort was measured from the mean absolute value of the electromyography signals from a wrist flexor-extensor muscle pair of each partner normalized to their torque-equivalent values (see Experiments for calibration procedure). We computed the *interaction effort*, *α*_*c*_/*α* − 1, as the difference between the effort in the connected trial *α*_*c*_ and the effort in the proceeding solo trial *α*. This interaction effort is plotted, in Fig 2B, as a function of the relative error for the hard (red), medium (blue) and soft interactions (green), where the lines are the fits to the data using a linear mixed-effects model with the interaction effort as the dependent variable and the relative error and the log of the coupling stiffness as the predictors (see Experiments for details). The linear mixed-effects analysis revealed that the interaction effort is dependent on the partners’ relative difference in performance (χ^2^(1) = 7.20, *P*<0.008), and it is also linearly modulated by the log of the coupling stiffness (χ^2^(2) = 27.2, *P*<10^−5^). We calculated the mean effort of the better and worse partners within each dyad for every level of coupling stiffness. A repeated-measures ANOVA, with a Greenhouse-Geisser correction for the violation of the sphericity assumption (Mauchly’s test, χ^2^(2) = 30.3, *P*<10^−6^), determined that the coupling stiffness significantly changed the mean effort (*F*(2,44) = 9.56, *P*<0.004). One-sample t-tests were conducted using a Bonferroni correction with a significance level of 0.05/6, which revealed that the mean interaction effort of the better partner during hard (*t*(12) = 4.21, *P*<0.0012) and medium interactions (*t*(12) = 3.52, *P*<0.0042) were different from zero. Hence, the better partner exerted more effort during the hard and medium interactions relative to when they did the task alone. This may be due to the better partner taking the lead of the movement, and counteracting the adverse movements made by the worse partner only when the coupling stiffness is strong enough to do so.

**Fig 3:**
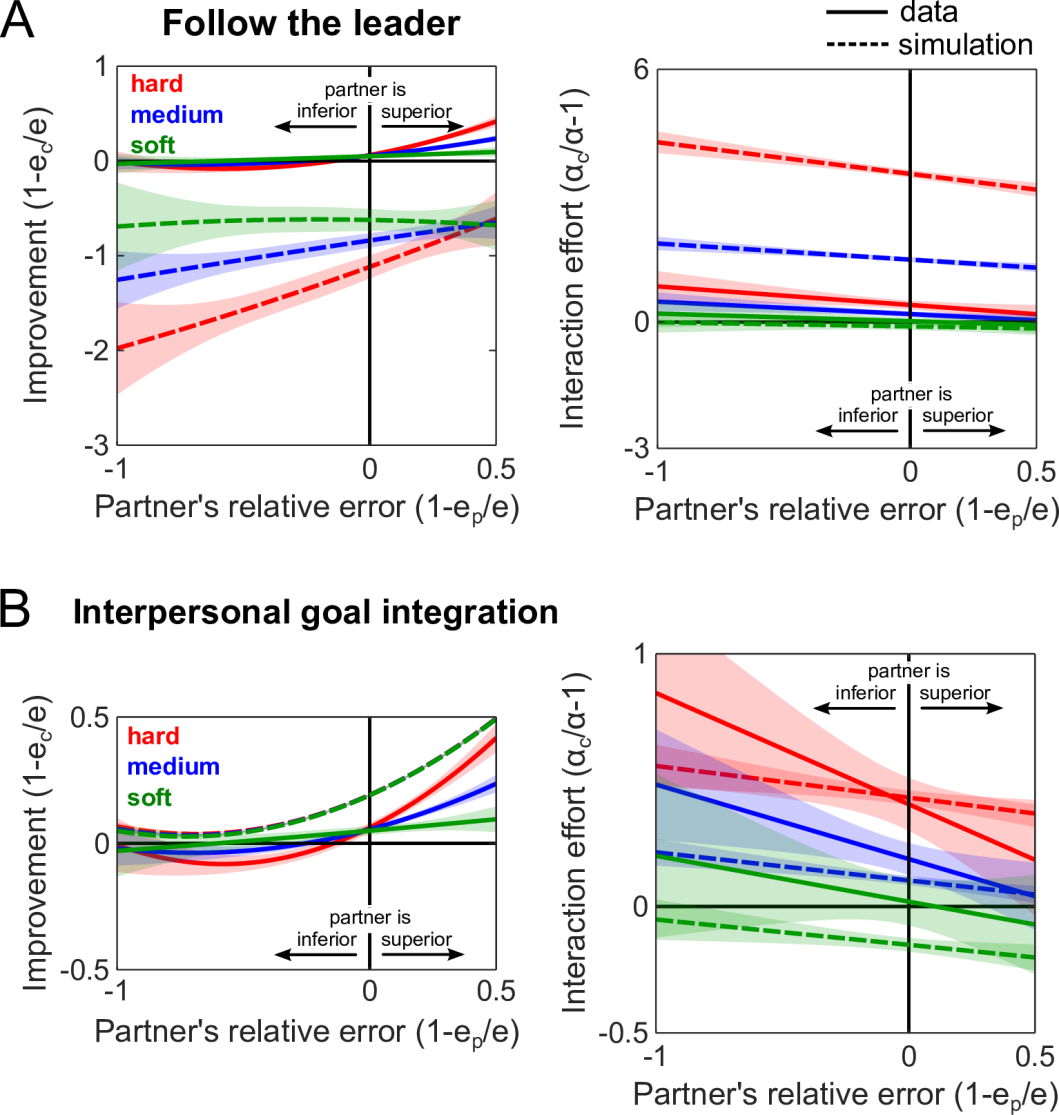
The follow the leader and interpersonal goal integration models cannot replicate the hard and soft interactions. (A) Prediction of the improvement and interaction effort from the *follow the leader* model, where subjects switch to following the partner’s cursor if they are nearest to the target. The prediction overestimates the interaction effort by a large amount, and greatly underestimates the performance improvement. (B) Predictions from the *interpersonal goal integration* model, where the visual and haptic information of the target are combined to yield a better prediction of the target’s motion. The haptic estimate of the target is obtained by forming a representation of the partner that identifies how the partner’s movement changes in response to the motion of the target, which is used to estimate the partner’s target. Unlike the data, this model does not exhibit any modulation in the improvement due to the coupling stiffness.

These results suggest that the coupling dynamics finely tune both the performance improvement and the interaction effort, and supports the hypothesis that the worse partner follows the lead of the better partner when the interaction is hard, which is consistent with the *follow the leader* model. However, partners may adopt a different behavior during soft interaction. To test whether a *follow the leader* model can explain the results of both the hard and soft interactions, we used a computational modelling framework of two partners tracking the same target to simulate the outcomes of the interaction and compared them with the empirical data (Fig 1B; see Computational framework to simulate interaction). This model was simulated by assuming that the haptic force provides an estimate of the relative position of the partner’s wrist. A subject can follow the partner’s wrist if it is nearer to the target than one’s own wrist or, otherwise, follow the target (see ‘follow the leader’ panel in Fig 1B). This model is inspired by decision-making studies where the best partner’s decision is taken by a group [23,24]. Surprisingly, this *follow the leader* model predicted performance detriment for both the better and worse partners, and greatly overestimated the effort exerted by both partners (see Fig 3A). Although this model was intuitively plausible, the simulations demonstrate that it cannot explain the outcomes of even the hard interaction.

In a previous study [18], we showed that human behavior during soft interactions can be explained by a model where partners share their movement goals with each other. Can this *interpersonal goal integration* model predict the experimental outcomes when the interaction is hard? In *interpersonal goal integration*, the haptic information from the physical coupling is used to form a representation of the partner that relates the motion of the target with the partner’s movement. This representation of the partner is used to estimate the partner’s goal, i.e. their target, which can be combined with one’s own visual estimate of the target. We simulated two partners who estimated each other’s goals in this manner (see ‘interpersonal goal integration’ panel in Fig 1B), but the improvement and interaction effort did not match the empirical data. Namely, the improvement was not graded as a function of the coupling stiffness as observed in the experimental data (Fig 3B, left panel). Furthermore, this model systematically underestimated the interaction effort (Fig 3B, right panel).

Although the *interpersonal goal integration* model cannot predict the improvement and the interaction effort during the medium and soft interactions, its predictions for the hard interaction resemble the results from the experiment. It should be noted that the coupling stiffness will filter the transmitted haptic forces and modify them, especially if the interaction is soft [6]. This may be a particularly important factor to consider in the context of the *interpersonal goal integration* model, where subjects must *estimate their partner’s goal through the mechanics of the entity that mediates the interaction*. Hard coupling will transmit the partner’s force faithfully, but soft mechanics filter the force relayed to the partner. Consequently, the estimate of the partner’s goal may be poor during soft interaction, and accurate when the interaction is hard.

**Fig 4:**
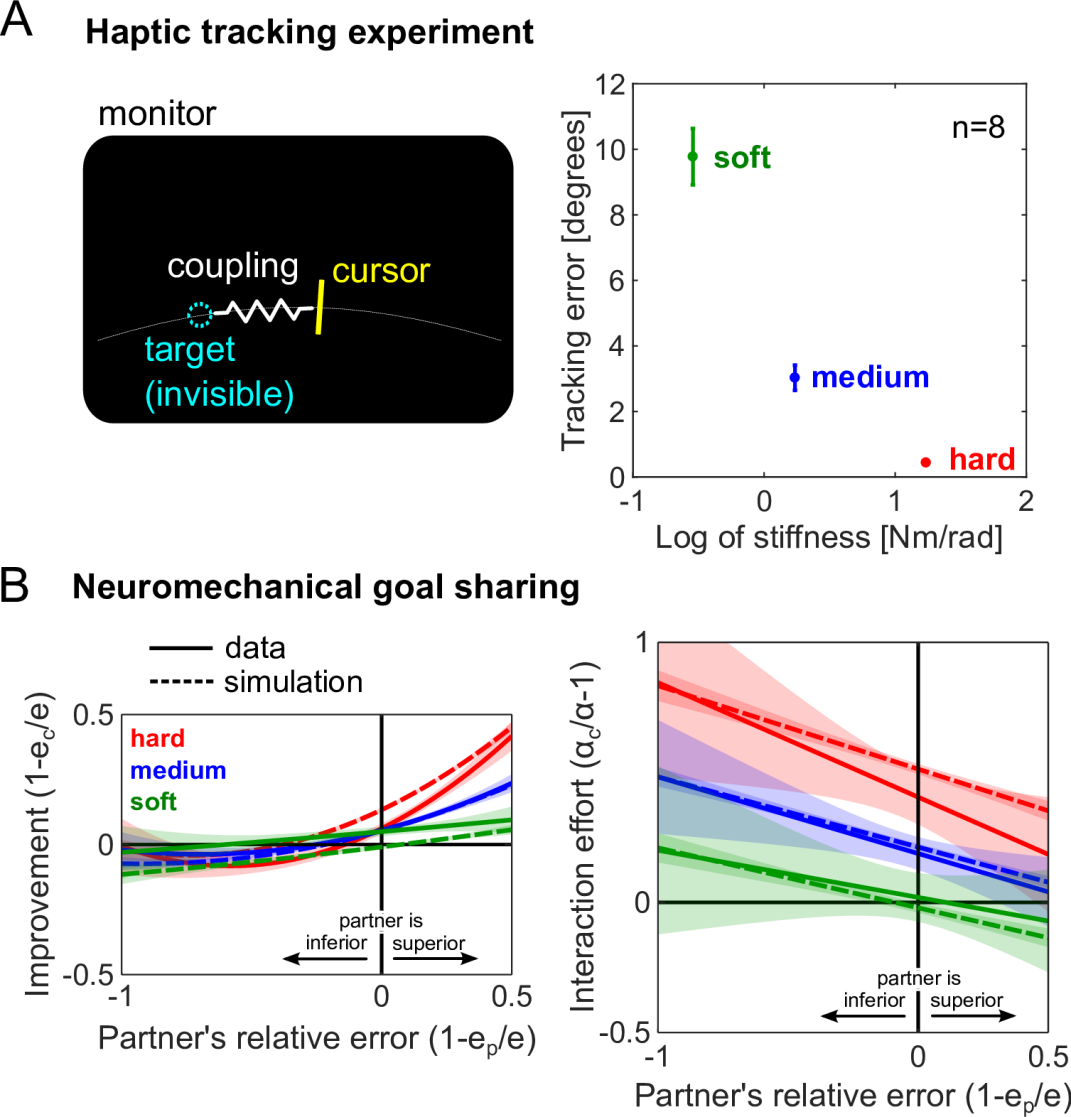
Coupling dynamics influence haptic communication. (A) Haptic tracking control experiment to measure how the tracking error, or the noise in the haptic information, increases with the coupling stiffness (plots are mean±standard error). 8 individually recruited subjects received no visual feedback of the target, instead relying only on the haptic feedback from the elastic connection between the wrist and the target. (B) The tracking errors from the haptic tracking experiment were considered in the *neuromechanical goal sharing* model, which combines information from vision with the haptic sensing of the partner’s goal to improve tracking performance while taking into account the additional noise in haptic sensing arising from the softness of the interaction. The predictions from this model closely resemble the data, exhibiting modulation of both the improvement and interaction effort due to the coupling stiffness. This shows that the effort expended during interaction is not a strategy per se, but the outcome of maximizing the sensory information of the target’s motion.

To characterize how the softness of the coupling dynamics influences the estimate of the partner’s goal, we conducted a *haptic tracking control experiment*. 8 subjects were recruited individually to track a target with visual feedback of the cursor, but not of the target (left panel of Fig 4A). Subjects received only haptic feedback through a virtual elastic band that connected the subject’s wrist to the target. By measuring the tracking error as a function of the stiffness of the elastic band, we could assess how the estimation of the target’s motion changes with the coupling stiffness. The haptic tracking errors are shown as a function of the log of the coupling stiffness in the right panel of Fig 4A. Indeed, the softer interaction resulted in poorer estimation of the target’s movement, yielding larger tracking errors.

This extra error arising from the coupling dynamics can be used to modify the *interpersonal goal integration* model. In this novel *neuromechanical goal sharing* model (see Interaction strategies), the softness of the interaction is assumed to add extra noise to the haptic measurement of the partner’s goal. The predictions of the *neuromechanical goal sharing* model for the improvement and interaction effort are shown in Fig 4B. The predictions for both the improvement and the interaction effort correspond well to the data (see S1 Text for a rigorous comparison of the different models of interaction). The performance is correctly modulated by the coupling stiffness, which influences the estimate of the partner’s goal and, in turn, increases the effort necessary to carry out the tracking task successfully. Critically, the *neuromechanical goal sharing* model can explain a human’s behavior during both hard and soft interactions.

## Discussion

We systematically investigated how pairs of human subjects tracked a 1 dimensional target motion using the flexion-extension of the wrists, which were coupled by a virtual elastic band with different stiffness. By measuring the change in each partner’s muscular activity during this interactive task, we could identify how each person’s effort depends on the other partner’s relative skill, and how the stiffness of the elastic band modulates this relationship. Furthermore, measuring the tracking performance on coupled relative to uncoupled trials as a function of the coupling stiffness provided insights into how the coupling dynamics affects each partner’s accuracy at tracking the target. This experimental approach might in future allow us to examine how partners physically cooperate in scenarios ranging from a physiotherapist assisting a patient to workers moving a table or a mattress together.

Our experiments revealed that the performance and the effort were tuned as a function of both the coupling stiffness and the partners’ individual skills. The worse partner’s performance improved more when the interaction with the better partner was stiffer. Importantly, the performance of the better partner was not hindered during the hard interaction with a worse partner, but this came at the cost of the better partner having to exert more effort than when doing the task alone. The increasingly larger effort exerted by the better partner during the medium and hard interactions suggested that the assignment of leadership [1–5] could explain the experimental results. However, the predicted outcomes of the computational simulations of this *follow the leader* model were starkly different from the data for both the soft and even the hard interactions.

The results can be better explained by the *interpersonal goal integration* model, proposed in a previous study [18], where the partners share their movement goals through the interaction forces. However, the best model accounted for the mechanics of the interaction between the partners and understand how it influences the partners’ performance and effort. Specifically, we showed how the haptic sensing is affected by the coupling dynamics in a haptic tracking experiment, and integrated this relationship into our model. This *neuromechanical goal sharing* model makes best use of the haptic information provided by the partner *through the coupling dynamics* to anticipate the target’s movement. It predicts the trends observed in the performance improvement and interaction effort as a function of the relative difference in the partners’ task performance, and their modulation due to the coupling stiffness. It should be noted that the *neuromechanical goal sharing* model outperforms all other models tested in this study, but there are discrepancies between its predictions and the empirical data. This could be due to a variety of reasons, such as the variability that arises from two human subjects interacting and adapting to one another, or could be due to the assumption that all subjects experience the same overall reduction in the ability to estimate the partner’s goal. A less variable measure of the interaction effort may yield further insights into the effect of the coupling dynamics on interaction behavior.

The *interpersonal goal integration* model was able to explain the compliant interaction in our previous study [18], but not so during this task. This discrepancy is due to the limited data in our previous study where only the performance improvement for the medium interaction was measured, and not the interaction effort. The *interpersonal goal integration* model also reproduces the medium interaction in this study, but only if we ignore the hard and soft interactions and the interaction effort (see S2 Fig in S1 Text). Our new study provides additional empirical data for hard and soft interactions, and the muscular effort of each partner, that enabled us to comprehensively investigate the limits of the *interpersonal goal integration* model, and deepened our understanding of the goal sharing coordination mechanism.

Altogether, the experimental results and the computational modelling suggest that the coupling dynamics may determine the amount of information that can be communicated by physically interacting partners. In this regard, the central nervous system is aware of the coupling dynamics and its effect on the information from the interaction force [6], and weighs the sensory measurements from vision and haptics according to their noise [19]. This can be considered as a form of morphological computation where the dynamics of the interaction of the limb and the environment are considered by the brain [25,26] to generate appropriate motor commands.

In comparison to other models, the *neuromechanical goal sharing* model could predict the trends in the human behavior of two subjects accomplishing the same task together. However, what happens in different interactive tasks where multiple solutions [27] can be taken, or when the partners must compete with one another? In such scenarios, it is still useful to estimate the partner’s goal, but it would not be integrated with one’s own goal. Instead, a subject could plan their movement to accomplish their task whilst deliberately avoiding the partner’s goal. The ability to estimate the partner’s goal may be a common factor in all interactive tasks, and the collaborative or competitive nature of the task [28] could determine how subjects use this information to their advantage.

## Materials and methods

### Ethics statement

The experiments were performed at the Department of Bioengineering at Imperial College London. The participants gave informed consent for their participation in the experiments, which were conducted according to the principles in the Declaration of Helsinki and approved by the Imperial College Research Ethics Committee.

### Experiments

A one degree of freedom, dual robotic interface shown in Fig 1A was used for the experiment [22]. Subjects controlled a cursor on a computer monitor using their wrist flexion/extension to track a moving target, which was composed of a multi-sine function of the form

t(τ)=−7.8sin⁡(0.12τ)+1.6sin⁡(0.28τ)+9.4sin⁡(0.37τ)−10.6sin⁡(0.64τ)+η (1)

where *τ* is the time and $\eta \in G(0,\sigma_{t})$ is Gaussian noise with standard deviation *σ*_*t*_ that was fixed throughout the experiment, and was different for each subject. This visual noise was implemented through ten normally distributed spots about the target, creating a ‘cloud’ which was updated every 400ms, and the standard deviation of the spots was kept constant through the experiment. The target movement was randomized through the selection of the initial time according to a uniform stochastic distribution in the interval between 0–30 seconds.

The *tracking error* was taken as the root-mean squared distance between the cursor and the target over a trial. 28 subjects were asked to complete the tracking task in 9 male-male and 5 female-female pairs, who were separated by a curtain during the experiment to mitigate the influence of social factors [21]. The experiment consisted of 45 trials, each trial lasting 40s. Connected trials, during which dyads were coupled through a virtual elastic band, were selected by a 50% dice throw. The experiment was split into 3 blocks of 15 trials where dyads experienced a different coupling stiffness *K* in each block of either 17.2 Nm/rad (*hard interaction*), 1.7 Nm/rad (*medium interaction*) or 0.3 Nm/rad (*soft interaction*). Dyads always experienced the medium interaction first. Thereafter, the order of the hard and soft interactions was randomized.

Surface electromyography (EMG) was used to collect data from the flexor carpi radialis (FCR) and extensor carpi radialis longus (ECRL) muscles. Subjects were asked to flex or extend their wrist while the device kept their wrist locked at 0 degrees, the subject’s most comfortable position. The subject was asked to produce flexion and extension torques of 1, 2, 3 and 4 Nm for 2 seconds, first flexion then extension, with a rest period of 5 seconds between each activation to prevent fatigue. This EMG data was linearly regressed versus torque to estimate the relationship between muscular activity and torque. The effort measure used in this study uses these estimated torques values.

Another 8 subjects were recruited individually to carry out a *haptic tracking control experiment*. The target movement was the same as in Eq (1) and the cursor was visible but the target was invisible through the trial. However, the subject was physically coupled to the target via a spring with the same stiffness as the hard, medium and soft interactions to receive haptic feedback of the target’s motion. Subjects tracked the haptic target for 40s, and experienced 5 trials of each coupling stiffness consecutively in the order of the hard, medium and soft interactions, respectively.

A linear mixed-effects model was employed to fit the improvement Δ*c* = 1 – *e*_*c*_/*e*, where *e* is the error of a subject in a solo trial and *e*_*c*_ is the same subject’s error on a connected trial, as a function of the relative error, Δ*p* = 1 – *e*_*p*_/*e*, where *e*_*p*_ is the partner’s error in a solo trial, and the log of the coupling stiffness *κ* = log_10_(*K*):

$$∆c=\beta_{0\rho }+\beta_{1\rho }∆p+\beta_{2\rho }\kappa +\beta_{3\rho }{∆p}^{2}+\beta_{4\rho }(∆p∙\kappa )+\beta_{5\rho }({∆p}^{2}∙\kappa )+\varepsilon_{\rho }$$ (2)

where *β*_0*ρ*_ is the intercept, *β*_1*ρ*_ to *β*_5*ρ*_ are the parameters for each predictor and 03 *ε*_*ρ*_ is the unexplained variance of the improvement for each dyad *ρ*.

A similar linear model was used to fit the effort data with the interaction effort *E* = *α*/*α*_*c*_ − 1, where *α* is the effort in a solo trial and *α*_*c*_ is the effort in a connected trial, as a function of the dependent variable, the relative error Δ*p* and *κ* as predictors:

$$E=\beta_{0\rho }+\beta_{1\rho }∆p+\beta_{2\rho }\kappa +\beta_{3\rho }(∆p∙\kappa )+\varepsilon_{\rho }.$$ (3)

In both of these linear models, we compared each model without the coupling stiffness term *κ* and their interaction terms using a likelihood ratio test to demonstrate the significance of the coupling dynamics on the performance improvement and the interaction effort. The same test was conducted by replacing the predictor *K* with a categorical variable that labelled whether the dyads experienced the medium, hard then soft interactions or the medium, soft then hard interactions. This revealed that neither the performance improvement (χ^2^(3) = 2.53, *P*>0.47) nor the interaction effort (χ^2^(2) = 0.48, *P*>0.79) were affected by the ordering of the coupling stiffness that dyads experienced. Therefore, all dyads’ data were analyzed together.

### Computational framework to simulate interaction

A model was developed in discrete time {*i dt*, *i* = 1,2,…} to simulate how two partners coupled by an elastic band plan their movement to track a randomly moving target in 1 dimension. At every time index *i* the target with position *t*_*i*_ must be estimated, then a motor command *u*_*i*_ is generated to move the wrist’s position *θ*_*i*_ to the target. First, we describe the state equation that governs the movement of the target, and then that of the wrist, and combine these two equations to formulate a single state equation of the full system. The movement of the target, which is assumed to be moving randomly via Gaussian noise in its velocity ${\dot{t}}_{i}\equiv \mu_{i}$, is described by the first-order system

$$t_{i+1}=At_{i}+\left[\begin{array}{c} 0\\ dt \end{array}\right]\mu_{i},\;A\equiv \left[\begin{array}{cc} 1 & dt\\ 0 & 1 \end{array}\right],\;\mu_{i}\in G(0,M_{i})$$ (4)

where $t_{i}\equiv {\left[t_{i},{\dot{t}}_{i}\right]}^{T}$ is the *target state* and $M_{i}\equiv E[t_{i}\;t_{i}^{T}]$ is the covariance matrix.

The control of the wrist, with state $\theta_{i}\equiv {\left[\theta_{i},{\dot{\theta }}_{i}\right]}^{T}$, is modelled as $I{\ddot{\theta }}_{i}=u_{i}+F_{i}$ with inertia *I*, i.e. in state-space format as

$$\theta_{i+1}=A\theta_{i}+B(u_{i}+F_{i}),\;B\equiv \left[\begin{array}{c} 0\\ dt/I \end{array}\right]$$ (5)

where

$$u_{i}=-[L^{p},L^{v}](\theta_{i}-t_{i})$$ (6)

which is the control law to move the wrist towards the target, and *F*_*i*_ is the force from the elastic band, with stiffness *K* and damping *D*. The partner’s wrist, described by the state ${\tilde{\theta }}_{i}\equiv {\left[{\tilde{\theta }}_{i},{\tilde{\dot{\theta }}}_{i}\right]}^{T}$ is connected to one’s own wrist through the elastic band that produces an elastic force,

$$F_{i}=K({\tilde{\theta }}_{i}-\theta_{i})+D({\tilde{\dot{\theta }}}_{i}-{\dot{\theta }}_{i}).$$ (7)

Solo trials, where the subjects are not connected, are characterized by zero force *F*_*i*_ ≡ 0.

A subject using the motor command as described in Eq (6) to move their wrist according to Eq (5) to follow the target that moves according to Eq (4) is described by the full state equation

$$x_{i+1}=Ax_{i}+B(u_{i}+F_{i}+\mu_{i}I)$$ (8)

which is equivalent to the difference of Eq (5) minus Eq (4).

### Interaction strategies

Various interaction strategies are described from the sensory information exchange between the partners. First, we describe the behavior of one subject tracking the target *t*_*i*_ alone using only visual feedback. To generate the motor command according to Eq (6), the state describing the difference between the target and the wrist is observed through

$$z_{i}=\theta_{i}-t_{i}+\nu_{i}$$ (9)

where the observation *z*_*i*_ is corrupted by Gaussian visual sensory noise *ν*_*i*_ with variance $\sigma_{v}^{2}\equiv E[{\left(z_{i}-E[z_{i}]\right)}^{2}]$. This linear quadratic estimation is computed in discrete time using an iterative Kalman filter algorithm [29]. Sensory delay in vision and proprioception is compensated by integrating Eq (8).

Now that we have described how to visually track a target, what motion planning model could be used to track the randomly moving target whilst being physically coupled to a partner? In the *follow the leader* model that subjects follow the partner’s wrist position ${\tilde{\theta }}_{i}$ if it is nearer to the target, otherwise the target’s position *t*_*i*_ is tracked. We assume that the state of the partner’s wrist ${\tilde{\theta }}_{i}$ is known to a subject from the interaction force in Eq (7). The observation is dependent on

$$z_{i}=\left\{ \begin{array}{cc} \theta_{i}-t_{i}, & \left|\theta_{i}-t_{i}|\leq |{\tilde{\theta }}_{i}-t_{i}\right|\\ \theta_{i}-{\tilde{\theta }}_{i}, & \left|\theta_{i}-t_{i}|>|{\tilde{\theta }}_{i}-t_{i}\right| \end{array}\right..$$ (10)

The *interpersonal goal integration* model proposes that the partner’s target ${\tilde{t}}_{i}$ is optimally combined with one’s own visual estimate of the target *t*_*i*_, such that

$$z_{i}=\left[\begin{array}{c} \theta_{i}-t_{i}\\ \theta_{i}-{\tilde{t}}_{i} \end{array}\right].$$ (11)

The method to estimate the partner’s target ${\tilde{t}}_{i}$ from the interaction force with the partner and own vision and proprioception is described in a previous paper [18]. First, one considers the partner’s control law

$${\tilde{u}}_{i}=-[{\tilde{L}}^{p},{\tilde{L}}^{v}]\left[\begin{array}{c} {\tilde{\theta }}_{i}-{\tilde{t}}_{i}\\ {\tilde{\dot{\theta }}}_{i}-{\tilde{\dot{t}}}_{i} \end{array}\right],$$ (12)

similar to one’s own control law Eq (6). To estimate the partner’s target, the partner’s control gain $\tilde{L}\equiv [{\tilde{L}}^{p},{\tilde{L}}^{v}]$ must be identified. To do so, Eq (12) must be inverted; the state of the partner’s wrist $\tilde{\theta }$ is estimated through the interaction force *F*_*i*_, the partner’s motor command ${\tilde{u}}_{i}$ is estimated through the changes in the state of the partner’s wrist, and the partner’s target is initially substituted with one’s own target state **t**_*i*_. Thus, to identify the partner’s control gain, the following sensory information must be observable:

$$z_{i}=\left[\begin{array}{c} \theta_{i}\\ t_{i}\\ F_{i} \end{array}\right]$$ (13)

where one’s own wrist position *θ*_*i*_ is measured through proprioception, one’s own target position *t*_*i*_ is measured through vision and the interaction force *F*_*i*_ is measured through haptics. In discrete time, the representation of the partner to estimate their control policy can be described by the equations:

$$\theta_{i+1}=\theta_{i}+{\dot{\theta }}_{i}dt,\;{\dot{\theta }}_{i+1}={\dot{\theta }}_{i},\;t_{i+1}=t_{i}+{\dot{t}}_{i}dt,\;{\dot{t}}_{i+1}={\dot{t}}_{i}$$

$${\tilde{\theta }}_{i+1}={\tilde{\theta }}_{i}+{\tilde{\dot{\theta }}}_{i}dt,\;{\tilde{\dot{\theta }}}_{i+1}={\tilde{\dot{\theta }}}_{i}+\left[-{\tilde{L}}_{i}^{p}({\tilde{\theta }}_{i}-t_{i})-{\tilde{L}}_{i}^{v}({\tilde{\dot{\theta }}}_{i}-{\dot{t}}_{i})\right]dt$$ (14)

$${{\tilde{L}}_{i+1}^{p}={\tilde{L}}_{i}^{p},\;{\tilde{L}}_{i+1}^{v}={\tilde{L}}_{i}^{v},\;F}_{i+1}=K({\tilde{\theta }}_{i}-\theta_{i})+D({\tilde{\dot{\theta }}}_{i}-{\dot{\theta }}_{i})$$

While identifying the partner’s control policy $\tilde{L}$, one can infer the partner’s target by replacing one’s own target **t** in Eq (14), which was initially substituted in the beginning, with the partner’s target $\tilde{t}$, yielding

$${\tilde{t}}_{i+1}={\tilde{t}}_{i}+{\tilde{\dot{t}}}_{i}dt,\;{\tilde{\dot{t}}}_{i+1}={\tilde{\dot{t}}}_{i},\;{\tilde{\dot{\theta }}}_{i+1}={\tilde{\dot{\theta }}}_{i}+\left[-{\tilde{L}}_{i}^{p}({\tilde{\theta }}_{i}-{\tilde{t}}_{i})-{\tilde{L}}_{i}^{v}({\tilde{\dot{\theta }}}_{i}-{\tilde{\dot{t}}}_{i})\right]dt$$ (15)

and with sensory information from only proprioception and haptics,

$$z_{i}=\left[\begin{array}{c} \theta_{i}\\ F_{i} \end{array}\right].$$ (16)

Eqs (13)–(16) enable one to both estimate the partner’s control policy and infer the partner’s target. Note that the partner’s target can be estimated from knowledge of their control policy and from proprioceptive and haptic sensory information only; the substitution with one’s own target is only necessary to identify the partner’s control policy.

The *neuromechanical goal sharing* model suggests that partners integrate vision and haptics as in Eq (11), but the haptic information is degraded by the coupling stiffness, which is modelled as additive sensory noise. What is the sensory noise in the haptic measurement of the partner’s target? We assumed that the haptic noise is equivalent to the partner’s visual noise in the tracking task ${\tilde{\sigma }}_{v}^{2}$ plus a noise due to the softness of the coupling dynamics $\sigma_{s}^{2}$. The value of $\sigma_{s}^{2}$ for each coupling stiffness was taken from the haptic tracking control experiment (see S1 Text for more information).

### Sensitivity analysis

For each proposed model, we conducted a sensitivity analysis to compare their predictive power over a parameter space. As the length of the trial is sufficiently long and the tracking task is continuous, we used an infinite-horizon optimal controller with quadratic cost. Two parameters of the model are adjustable; $\sigma_{\mu }^{2}$, a multiplier for the Gaussian noise *μ*_*i*_ in the acceleration, and *q* > 0, a multiplier for the state cost **Q** in the controller. **L** will minimize the cost functional

$$J\equiv \sum_{i=1}^{\infty }x_{i}^{T}(qQ)x_{i}+u_{i}^{T}Ru_{i}$$ (17)

where **x**_***i***_ is the combined state vector, *u*_*i*_ is the motor command, the state cost *q***Q** is positive semi-definite and control cost *R* > 0.

$\sigma_{\mu }^{2}$ is a scaling factor of the process noise that determines the frequency content of the simulated trajectory, and *q* modulates the strength of the partners’ controllers. $\sigma_{\mu }^{2}$ was bound within a range such that the trajectories were not jerky, and *q* was varied within a stable controllable range (see S1 Text).

The root mean squared error was used as a metric for predictive power. Over the whole parameter space, the *neuromechanical goal sharing* model had the most predictive power (S1 Fig in S1 Text).

## Supporting information

S1 FigSensitivity analysis of the interaction models for the two parameters in the simulation.The log of the MAE between the fits from the data and the simulation as a function of *q* and $\sigma_{\mu }^{2}$. The MAE is relatively insensitive to changes in the strength *q* and $\sigma_{\mu }^{2}$. The *neuromechanical goal sharing* model has a lower RMSE than the *follow the leader* and *interpersonal goal integration* models for all parameter values.(TIF)

S2 Fig Interpersonal goal integration model reproduces the medium interaction, but only if the other conditions and the interaction effort are ignored. In a previous study [18], we showed that the *interpersonal goal integration* model reproduced the medium interaction performance improvement. It can also reproduce the medium interaction data in this study, but only if the hard and soft interactions and the interaction effort, are ignored. Thus, the additional empirical data from this new study has refined our understanding of the goal sharing mechanism, resulting in the *neuromechanical goal sharing* model where the coupling dynamics influence the estimation of the partner’s goal.
(TIF)

S1 Text This document describes the sensitivity analysis used to find the simulation parameters for the best fit to the data for each mechanism. We also demonstrate that the *interpersonal goal sharing* model from our previous study can explain the medium performance improvement, but only if the hard and soft interactions and the interaction effort are ignored. Thus, this model was sufficient for the previous study that only examined performance improvement during medium interaction, but cannot explain the interaction with different coupling dynamics.
(DOCX)

S1 Data Text file containing data from all 14 dyads in the human-human interaction experiments. Each row comes from a paired data point that compares a connected trial with the proceeding solo trial. Each column contains data in the following order where 1 denotes one partner and 2 the other: solo error (1), solo error (2), connected error (1), connected error (2), solo total effort (1), solo total effort (2), connected total effort (1), connected total effort (2), solo co-contraction (1), solo co-contraction (2), connected co-contraction (1), connected co-contraction (2), solo reciprocal activation (1), solo reciprocal activation (2), connected reciprocal activation (1), connected reciprocal activation (2), connection stiffness, dyad number, and order of connection blocks. Errors are in degrees, effort measures are all in N/m and stiffness in Nm/deg. The order of connection is 1 for medium, hard and soft and 0 for medium, soft and hard.
(TXT)

S2 Data Text file containing data from all 8 subjects in the haptic tracking experiment. The data is composed of 30 rows and 8 columns, where the first 15 rows are the tracking error in degrees and the last 15 rows denote the connection stiffness of the respective trial in Nm/deg. Each column is from a separate subject.
(TXT)
