# Supplementary figures and images for: Haptic communication between humans is tuned by the hard or soft mechanics of interaction
Source: PLoS Comput Biol. 2018 Mar 22;14(3):e1005971. doi: 10.1371/journal.pcbi.1005971 (PMC5863953; doi:10.1371/journal.pcbi.1005971)

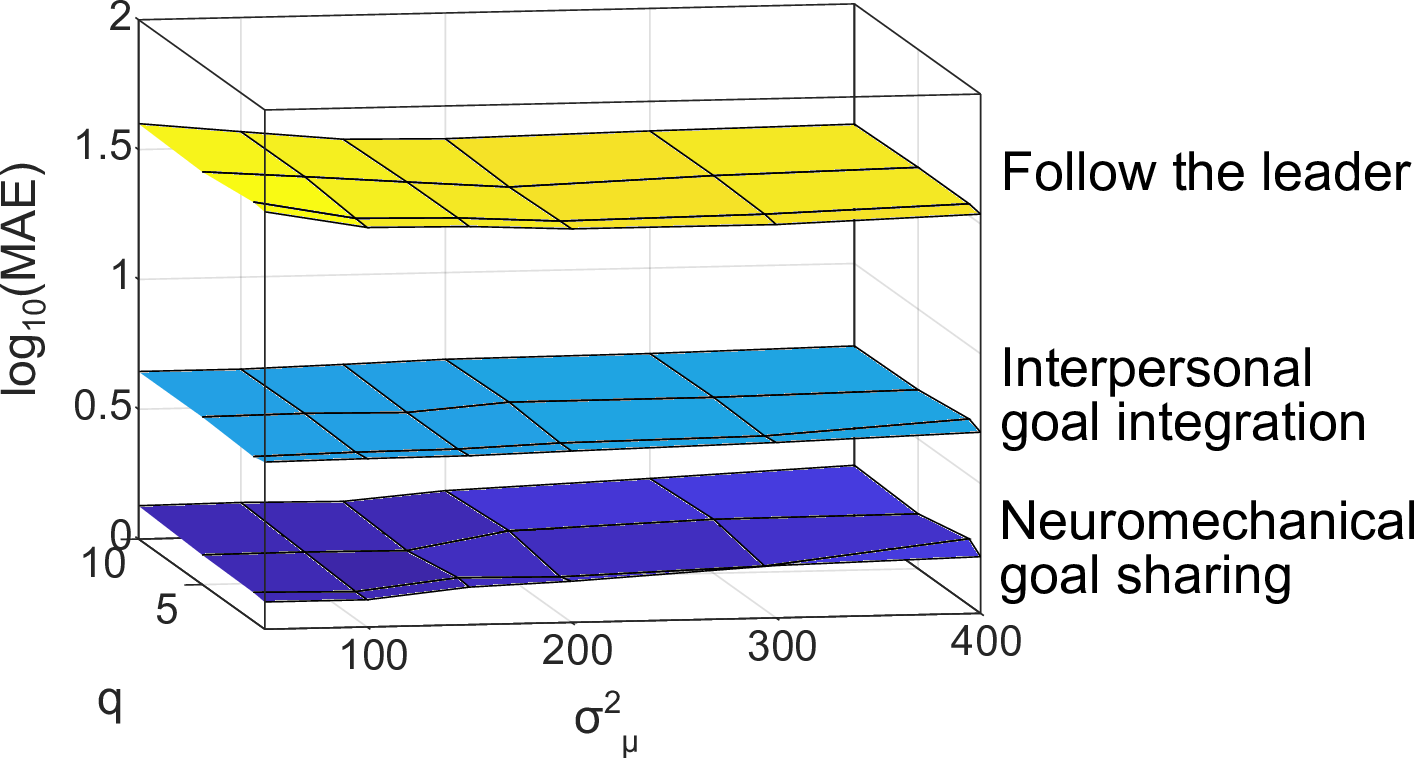

Supplement: S1 Fig — The log of the MAE between the fits from the data and the simulation as a function of q and σμ2. The MAE is relatively insensitive to changes in the strength q and σμ2. The neuromechanical goal sharing model has a lower RMSE than the follow the leader and interpersonal goal integration models for all parameter values. (TIF) [file pcbi.1005971.s001.tif]

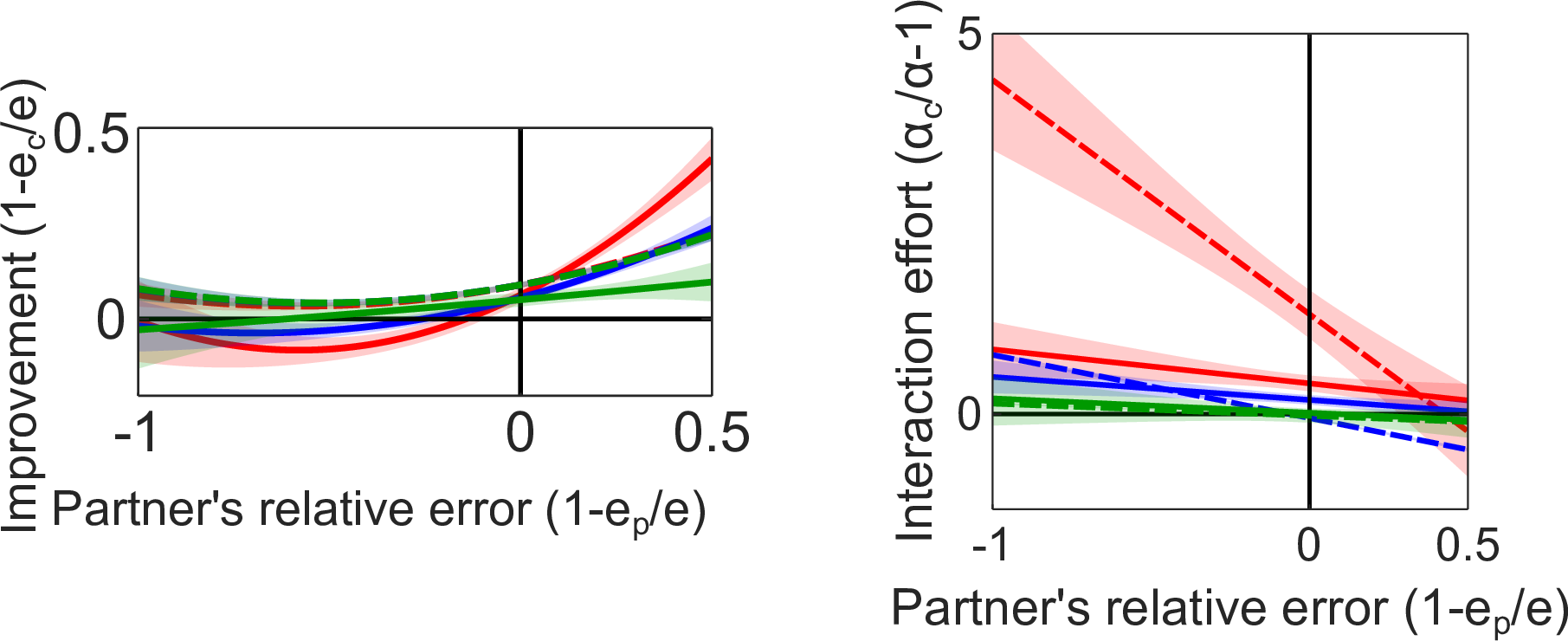

Supplement: S2 Fig — In a previous study [18], we showed that the interpersonal goal integration model reproduced the medium interaction performance improvement. It can also reproduce the medium interaction data in this study, but only if the hard and soft interactions and the interaction effort, are ignored. Thus, the additional empirical data from this new study has refined our understanding of the goal sharing mechanism, resulting in the neuromechanical goal sharing model where the coupling dynamics influence the estimation of the partner’s goal. (TIF) [file pcbi.1005971.s002.tif]
