# Supplementary material for: Haptic communication between humans is tuned by the hard or soft mechanics of interaction
Source: PLoS Comput Biol. 2018 Mar 22;14(3):e1005971. doi: 10.1371/journal.pcbi.1005971 (PMC5863953; doi:10.1371/journal.pcbi.1005971)
Supplement: S1 Text — We also demonstrate that the interpersonal goal sharing model from our previous study can explain the medium performance improvement, but only if the hard and soft interactions and the interaction effort are ignored. Thus, this model was sufficient for the previous study that only examined performance improvement during medium interaction, but cannot explain the interaction with different coupling dynamics. (DOCX) [file pcbi.1005971.s003.docx]

**Supplementary Information**

To calculate the predictive power of each model over the parameter space of *q* and $\sigma_{\mu}^{2}$, we employed the metric of the mean normalised absolute error (MAE), defined as the distance between the vector of points generated by the simulation $\boldsymbol{y}_{i}$ and the fit from the data ${\hat{\boldsymbol{y}}}_{i}$,

$\mathrm{MAE}=\frac{1}{n+1}\sum_{i=0}^{n} \frac{\left| \boldsymbol{y}_{i}-{\hat{\boldsymbol{y}}}_{i} \right|}{\boldsymbol{\sigma}_{\hat{y}}}$ (S1)

where $\boldsymbol{\sigma}_{\hat{y}}$ is the vector of standard deviations in the fitted data. The MAE was calculated for the improvement and interaction effort at each coupling stiffness level, yielding six values for the MAE for each unique pair of *q* and $\sigma_{\mu}^{2}$. The mean of the six values was taken as the final MAE value.

Two parameters were analyzed in our sensitivity analysis: the magnitude of the Gaussian noise in the target’s jerk $\sigma_{\mu}^{2}$, and the state cost multiplier *q*. $\sigma_{\mu}^{2}$ was bounded maximally <500 to avoid jerky trajectories. The other parameter *q* controls the human controller’s strength, which is bounded within realms of reasonable human strength between $0.01<q<1$. As the simulations in S1 Fig reveal, all strategies are insensitive to this parameter.

The sensory noise in the haptic information about the target coming from the partner through soft interaction dynamics is assumed to be the partner’s visual noise $\tilde{\sigma}_{v}^{2}$and some additive noise due to the interaction softness $\sigma_{s}^{2}$,

$\sigma_{h}^{2}=\tilde{\sigma}_{v}^{2}+\sigma_{s}^{2} .$ (1)

In the haptic tracking experiment (from S2 Data), we measured the additional error in the tracking task that arises due to the softness of the elastic spring. These error values can be used to estimate $\sigma_{s}^{2}$, and so the haptic noise is described by

$\sigma_{h}^{2}=\tilde{\sigma}_{v}^{2}+f(\sigma_{\mu}^{2},q,\tilde{\sigma}_{v}^{2},e,K)$ (2)

where f is a function that converts tracking error $e$ to an equivalent sensory noise. This function changes with respect to the process noise $\sigma_{\mu}^{2}$ as the performance of the Kalman filter is directly related to this value, and the controller strength $q$ that affects how closely one can follow the estimated target trajectory. To determine f, we simulated only the solo trials of the tracking task for each unique pair of $\sigma_{\mu}^{2}$ and $q$, and fitted a second order polynomial that related the standard deviation of one’s visual sensory noise $\sigma_{v}$ and tracking error $e$,

$\sigma_{v}=\gamma_{0}+\gamma_{1}e+\gamma_{2}e^{2}$ (3)

where $\gamma_{0}$, $\gamma_{1}$and $\gamma_{2}$ are fitted parameters. Since we assume that the softness of the interaction results in additive sensory noise, the haptic noise is

$\sigma_{h}^{2}={(\gamma_{0}+\gamma_{1}\left[ \tilde{e}+e_{k} \right]+\gamma_{2}{[\tilde{e}+e_{k}]}^{2})}^{2}$ (4)

where $\tilde{e}$ is the partner’s tracking error and $e_{k}$ is the additional error from the coupling stiffness$K$, whose values were taken from the haptic tracking experiment.

**
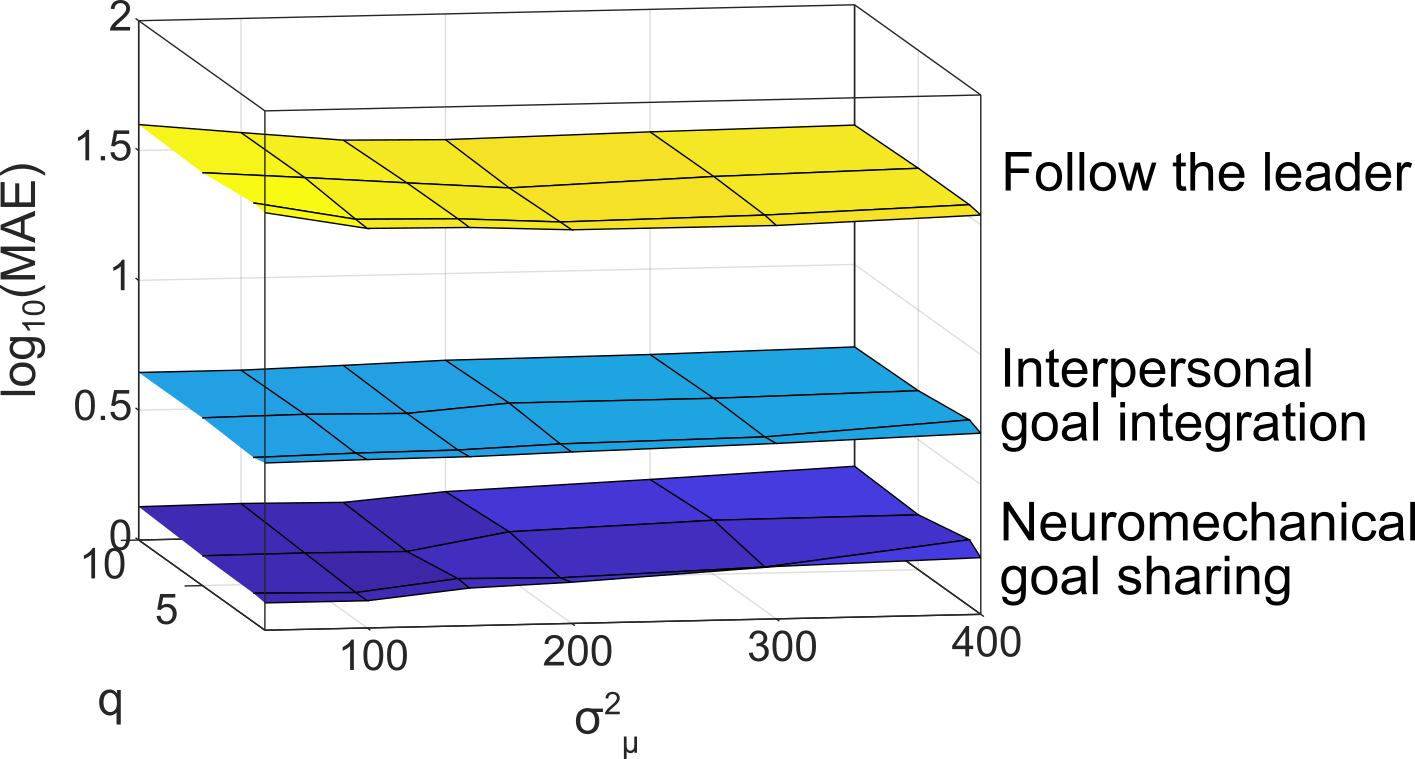
**

**S1 Fig Sensitivity analysis of the interaction models for the two parameters in the simulation.** The log of the MAE between the fits from the data and the simulation as a function of *q* and $\sigma_{\mu}^{2}$. The MAE is relatively insensitive to changes in the strength *q* and of $\sigma_{\mu}^{2}$. The *neuromechanical goal sharing* model has a lower RMSE than the *follow the leader* and *interpersonal goal integration* models for all parameter values.


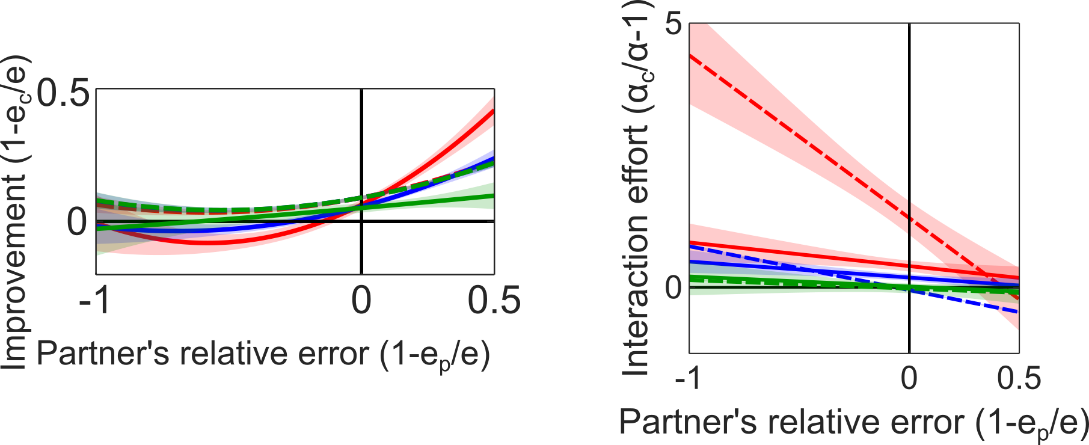


**S2 Fig Interpersonal goal integration model reproduces the medium interaction, but only if the other conditions and the interaction effort are ignored.** In a previous study [17], we showed that the *interpersonal goal integration* model reproduced the medium interaction performance improvement. It can also reproduce the medium interaction data in this study, but only if the hard and soft interaction conditions, and the interaction effort, are ignored. Thus, the additional empirical data from this new study has refined our understanding of the goal sharing mechanism, resulting in the *neuromechanical goal sharing* model where the coupling dynamics influence the estimation of the partner’s goal.
